# The Movement of Multidrug-Resistant Tuberculosis across Borders in East Africa Needs a Regional and Global Solution

**DOI:** 10.1371/journal.pmed.1001791

**Published:** 2015-02-24

**Authors:** Kevin P. Cain, Nina Marano, Maureen Kamene, Joseph Sitienei, Subroto Mukherjee, Aleksandar Galev, John Burton, Orkhan Nasibov, Jackson Kioko, Kevin M. De Cock

**Affiliations:** 1 United States Centers for Disease Control and Prevention, Kisumu and Nairobi, Kenya; 2 Kenya Ministry of Health, Nairobi, Kenya; 3 United States Agency for International Development, East Africa Regional Office, Nairobi, Kenya; 4 International Organization for Migration, Dadaab and Nairobi, Kenya; 5 United Nations High Commissioner for Refugees, Nairobi, Kenya

## Abstract

Kevin Cain and colleagues reflect on the cross border movement of people from Somalia with MDR-TB and the implications for MDR-TB programs in East Africa.

**Summary Points**

- Multidrug-resistant tuberculosis (MDR TB) and other deadly infectious diseases commonly occur in states suffering from political turmoil and armed conflict.
- The same conditions that promote MDR TB and other diseases often diminish the capacity of the public health system to address these needs, leading patients to seek care in other countries.
- In East Africa, a large number of patients from Somalia with MDR TB crossed the border to Kenya seeking treatment. While diagnostic capacity for MDR TB exists in Somalia, treatment capacity does not.
- Identification and management of such diseases need to be a priority for countries in the region both for humanitarian purposes and for the protection of their own residents. Often diseases will need to be diagnosed and treated outside of the country in which they are occurring.
- The solutions must be regional and global. Control of an infectious disease, such as MDR TB, must be focused at its source to be successful. Its control cannot depend on the existing capacity of the country in which it happens to occur.

## Background

Multidrug-resistant tuberculosis (MDR TB), or TB resistant to at least isoniazid and rifampin, is an important threat to global TB control [1]. Antituberculosis drug resistance, caused by inadequate or interrupted TB treatment, has been observed since the first clinical trials of anti-TB treatment were conducted in the 1940s. Emergence of drug resistance is promoted by ineffective TB control programs and interruption of drug supplies, often due to political and other social disruption. Following the collapse of the Soviet Union, MDR TB rose sharply in its constituent republics in the 1990s; some of those countries have the highest rates of MDR TB worldwide today [2–5].

Each year, approximately 480,000 people become ill with MDR TB, and 170,000 die [6]. While drug-susceptible TB can be cured in >95% of patients with 6 months of standardized treatment, treatment for MDR TB takes up to 2 years and succeeds in just 55%–67% of cases [3,7]. Even more concerning, 9.0% of the nearly half million MDR TB cases globally have extensively drug-resistant (XDR) TB, characterized by additional resistance to fluoroquinolones and at least one injectable anti-TB drug. MDR TB and XDR TB can cause outbreaks and pose an especially great threat to people with HIV [6]; an outbreak of XDR TB in people with HIV in South Africa killed 52 of the 53 people affected [7,8].

Currently, it is estimated that only about one in five MDR TB cases is diagnosed and an even smaller proportion are started on effective treatment [1]. The consequences of a large pool of untreated patients with MDR TB are well established. Many patients will die of their disease within 1–2 years, even though most deaths are avoidable with appropriate treatment. Untreated patients will spread MDR TB to family members and other contacts. Unabated transmission will cause the incidence of MDR TB to rise further [1].

### Kenya—A Sentinel Country Bordering Somalia

**Fig 1:**
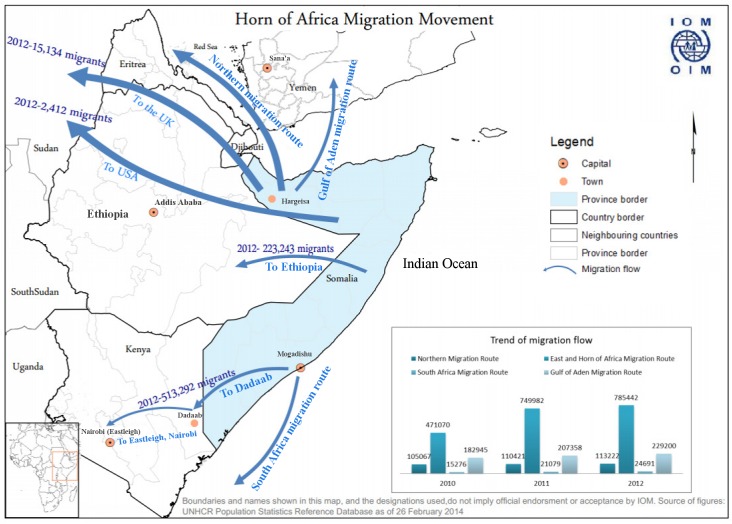
Map of Horn of Africa region showing key locations and also migration patterns for Somali refugees.

Kenya shares a long, porous border with Somalia, a country that has endured over two decades of civil strife, lack of an effective central government, breakdown of health services, and intermittent challenges, such as famine and outbreaks of polio. Dadaab, a small town in Eastern Kenya about 80 km from the Somali border, houses what is one of the world’s largest refugee camps, with over 400,000 predominantly Somali refugees housed in close proximity (Fig. 1) [9].

A large increase in MDR TB cases was reported in early 2013 in Dadaab, where 30 patients were diagnosed with MDR TB during the first quarter of 2013, compared to just ten in the same period of 2012. In July and August of 2013, MDR TB drugs ran short in Dadaab, and consequently, no new patients were started on treatment there. At the same time, Kenya’s TB program was notified of 16 cases of MDR TB in Eastleigh, a predominantly Somali area of Nairobi—a notable increase from its historical average of two cases per month.

Over 80% of Somali MDR TB patients diagnosed in 2013 in Dadaab and Eastleigh had come directly from Somalia rather than from within the refugee camps. Patients were reportedly arriving in Dadaab and Eastleigh from Somalia with the results of a GeneXpert test in hand showing rifampin-resistant TB and stating that they had MDR TB and had heard that treatment was available in Kenya.

### MDR TB in Somalia

In 2012–2013, five GeneXpert (personal communication, Dr. Subroto Mukherjee, United States Agency for International Development [USAID] East Africa) machines were placed in Somalia, including two in Mogadishu, but there was no capacity to treat patients with MDR TB. The Global Fund has allocated funds to scale up MDR TB treatment in Somalia, aiming to treat 60 patients by the end of 2014 and approximately 200 by the end of 2016, starting first in Hargeisa, in the semiautonomous region of Somaliland [10].

The burden of MDR TB, however, vastly exceeds the projected capacity. In a nationwide drug resistance survey in 2011, MDR TB was detected in 5.2% of all patients newly diagnosed with TB and in 40.8% of TB patients with a history of prior treatment, the highest documented rates of MDR TB on the African continent. An estimated 750 of the approximately 12,000 annual reported TB cases in Somalia have MDR TB [11]. The area in and around Mogadishu, which is nearer to Dadaab and Nairobi than Hargeisa (where MDR TB treatment is scheduled to be available), is especially affected [11].

### Regional and International Implications of MDR TB in Somalia

The challenge of MDR TB in Somalia has implications beyond Somalia’s borders. Over 400,000 Somali refugees currently reside in refugee camps within Kenya, with more living in other parts of Kenya; their movement within Kenya and across international borders is common. About 250,000 Somali refugees are in Ethiopia, and a similar number are in Yemen. Additionally, Somali refugees are relocated to many countries around the world, including the US and countries in Western Europe (Fig. 1) [12]. With ongoing MDR TB transmission, refugees can become latently infected with MDR TB and are then at risk for developing MDR TB later in life, which can initiate new transmission cycles in their new country of residence. This occurred among US-bound Hmong refugees in Thailand in 2006 and has also been described among Tibetan refugees in Canada and the US [13–16]. MDR TB has been reported previously to have crossed borders in Burmese persons in Thailand, Tibetan persons in India, and even in Somalis living in Kenya, among others [17–19].

## Strategies for Addressing the Problem

This is a multinational problem that requires a multinational response. The essential first step is to establish a principle that all patients with MDR TB should be treated, regardless of country of birth, citizenship, or permanent residence. In many cases, this will require patients to be treated outside of their home country. This represents not only the ethical approach to the regional challenge of MDR TB but enlightened self-interest for national health protection, as finding and initiating effective treatment in patients with MDR TB is essential to ending MDR TB transmission and therefore is an important priority for infection control [20]. For this to be effective, the principle needs to actually be implemented. With less than 25% of patients with MDR TB in most countries being treated [1], substantial scale-up is needed.

Next, we need to know when MDR is occurring, as currently about two-thirds of all MDR TB cases are never diagnosed [6]. This requires scale-up of MDR TB diagnostic capacity to meet estimated need and demand, as a long overdue public health imperative. Increasing diagnostic capacity will require (but should not be delayed while waiting for) an increase in treatment capacity. Introduction of GeneXpert in Somalia and Dadaab led directly to programmatic implementation of treatment for these patients. Had this capacity been delayed while waiting for diagnostic capacity to improve, the problem would have been neither detected nor mitigated. In the short term, this approach will result in diagnosis of more patients than can be treated. This can be partially resolved by ensuring that countries with MDR TB treatment capacity can help to provide treatment for those without such capacity, but its main solution will require more rapid effort to scale up MDR TB treatment capacity globally.

Third, the only long-term solution to ending the “push” that sends patients to other countries in search of life-saving treatment is to build capacity to treat and prevent MDR TB in Somalia. Since the majority of Somalis with MDR TB who came to Kenya came specifically for MDR TB treatment, provision of quality MDR TB care in Somalia is likely to limit cross border movement of patients with MDR TB. Likewise, efforts are urgently needed to prevent drug resistance in Somalia. This requires attention to the fundamentals of TB control, including optimizing the detection of patients with TB and ensuring that all are successfully treated, along with stopping transmission of MDR TB by detecting such patients and initiating effective treatment immediately [21]. Additionally, the supply of TB drugs within Somalia needs to be uninterrupted and regulated to ensure that the drugs are of high quality and are used properly.

Developing a strong and effective TB program in Somalia with the capability to manage and prevent MDR TB countrywide, while an essential goal, is one that is likely to take many years. Success will rely at least partially on the stability of the country, which in Somalia has been a challenge for decades. In the interim, it is essential to find ways to provide treatment for MDR TB patients in other countries while capacity is being built in Somalia. MDR TB diagnostic and treatment services and surveillance need to be strengthened in the East African region, especially in countries with large numbers of Somali refugees. The epidemic of MDR TB cases in Eastleigh when MDR TB treatment was unavailable in Dadaab is clear evidence that lack of availability of treatment in one location will result in migration of infectious patients elsewhere, often by crowded public transport. It is, therefore, both in the broader humanitarian interest and in the self-interest of each country to identify approaches by which patients from neighboring countries without MDR TB treatment capacity can be treated in countries with such capacity. Solutions require addressing the need for long durations of treatment and must account for the complexities of TB control in mobile populations [22,23].

## Challenges to Implementation and the Way Forward

There are several potential challenges that need to be addressed for the above approaches to successfully address the problem.

First, there is currently a lack of a regional funding mechanism to provide resources to address these regional needs. Currently, much of the funding for TB control in Africa comes from the Global Fund, which lacks a flexible, regional mechanism. Countries such as Kenya cannot use their own resources for diagnosis and treatment of MDR TB in refugees and migrants without an adverse effect on national TB program budgets, yet neither can they ignore the need to treat migrants from a humanitarian and public health perspective. For these reasons, we believe there is a need for a flexible, regional funding mechanism that would allow funds to be directed where the need—which can change rapidly—is greatest. Such a funding mechanism could come from large organizations like the Global Fund, from regional sources, or from other global donors, many of whom accept Somali and other refugees into their countries and have an interest in safely addressing the humanitarian needs of this population.

All of this will require political commitment to addressing MDR TB as a regional and global problem. The Kenya Ministry of Health, to its credit, has decided that all patients with MDR TB in Kenya must be treated, regardless of their nationality. We hope other countries in the region do the same. Neighboring countries with capacity to treat MDR TB should consider the benefits of scaling up that capacity to meet the needs of their own populations as well as of Somalis and nationals of other countries. Steps will need to be taken to ensure that treatment is uninterrupted. Globally, donor nations should carefully consider how they can help to address these needs. These actions represent not only the ethical approach to the regional challenge of MDR TB but enlightened self-interest for national health protection, as without identifying and treating patients with disease, there is no chance of mitigating the epidemic.

## Beyond East Africa and Beyond TB

Somalia reminds us that there are regional and global health and social consequences when states fail. The long experience of countries of the former Soviet Union with MDR TB provides a guide and stimulus for preventive actions to avoid further spread of drug resistance. Likewise, we must recognize that this threat is not unique to this region nor is it unique to TB. Other places exist in the world where conflict, collapse of political systems, natural disasters, and other factors simultaneously fuel the development of MDR TB or other communicable diseases, cripple the infrastructure needed to address it, and promote the migration of people in ways that causes it to spread. If we start by establishing and enforcing the right goal—that all patients with MDR TB or any other important communicable disease need to be identified and treated, regardless of birthplace or citizenship—we will be well placed to solve this and similar challenges. This example highlights the importance of dealing with infectious diseases at their source, even when the source countries lack the capacity to control a disease themselves. Doing so is not only the right thing to do for humanitarian reasons but is also essential to preventing unnecessary disease spread and deaths.
